# Growth and metabolic outcome in adolescents born preterm (GROWMORE): follow-up protocol for the Newcastle preterm birth growth study (PTBGS)

**DOI:** 10.1186/1471-2431-13-213

**Published:** 2013-12-20

**Authors:** Claire L Wood, Robert J Tinnion, S Murthy Korada, Timothy D Cheetham, Caroline L Relton, Richard J Cooke, Mark S Pearce, Kieren G Hollingsworth, Michael I Trenell, Nicholas D Embleton

**Affiliations:** 1 Child Health, Newcastle Hospitals NHS Foundation Trust, Newcastle upon Tyne NE1 4LP, UK; 2 Newcastle Neonatal Service, Newcastle Hospitals NHS Foundation Trust, Newcastle upon Tyne NE1 4LP, UK; 3 Institute of Health and Society, Newcastle University, Newcastle upon Tyne, UK; 4 Institute of Genetic Medicine, Newcastle University, Newcastle upon Tyne, UK; 5 MRC Integrative Epidemiology Unit, School of Social and Community Medicine, University of Bristol, Bristol, UK; 6 Institute of Cellular Medicine, Newcastle University, Newcastle Upon Tyne, UK

**Keywords:** Preterm birth, Insulin sensitivity, Childhood growth, Metabolic outcomes, Magnetic resonance spectroscopy

## Abstract

**Background:**

Preterm infants represent up to 10% of births worldwide and have an increased risk of adverse metabolic outcomes in later life. Early life exposures are key factors in determining later health but current lifestyle factors such as diet and physical activity are also extremely important and provide an opportunity for targeted intervention.

**Methods/Design:**

This current study, GROWMORE, is the fourth phase of the Newcastle Preterm Birth Growth Study (PTBGS), which was formed from two randomised controlled trials of nutrition in early life in preterm (24–34 weeks gestation) and low birthweight infants. 247 infants were recruited prior to hospital discharge. Infant follow-up included detailed measures of growth, nutritional intake, morbidities and body composition (Dual X Ray Absorptiometry, DXA) along with demographic data until 2 years corrected age. Developmental assessment was performed at 18 months corrected age, and cognitive assessment at 9–10 years of age. Growth, body composition (DXA), blood pressure and metabolic function (insulin resistance and lipid profile) were assessed at 9–13 years of age, and samples obtained for epigenetic analysis. In GROWMORE, we will follow up a representative cohort using established techniques and novel metabolic biomarkers and correlate these with current lifestyle factors including physical activity and dietary intake. We will assess auxology, body composition (BODPOD™), insulin resistance, daily activity levels using Actigraph™ software and use ^31^P and ^1^H magnetic resonance spectroscopy to assess mitochondrial function and intra-hepatic lipid content.

**Discussion:**

The Newcastle PTBGS is a unique cohort of children born preterm in the late 1990’s. The major strengths are the high level of detail of early nutritional and growth exposures, and the comprehensive assessment over time. This study aims to examine the associations between early life exposures in preterm infants and metabolic outcomes in adolescence, which represents an area of major translational importance.

## Background

The Developmental Origins of Health and Disease hypothesis suggest that nutritional imbalance during critical windows in early life can permanently influence long-term development and disease in later life [1]. Whilst the majority of epidemiological and other studies relate to infants born at term, there are increasing data, supported by controlled longitudinal studies, to show that similar effects occur in infants born preterm (<37 completed weeks gestation) [2]. Preterm births account for 5-10% of births in the UK and almost 10% of all births worldwide, representing more than 15 million births globally every year [3]. In developed countries, survival has dramatically improved in the last few years. More than 50% of babies born at 24 weeks gestation are now regularly surviving long term [4]. Despite these improvements, providing adequate nutrition to preterm infants is challenging and growth failure is common [5]. Most extremely preterm infants (<32 weeks gestation) require support with parenteral nutrition, metabolic ‘immaturity’ may limit nutrient intake, and enteral feeds take time to establish. Expressed breast milk is associated with a range of benefits in the short-term (e.g. reduction in the incidence of necrotising enterocolitis [6], a potentially fatal illness associated with milk feeds) and long-term (e.g. improved cognitive outcome), but alone will not meet nutrient requirements without fortification [7].

Because of these nutritional and other challenges, preterm infants accumulate nutrient deficits during hospital stay and most are discharged with weights below the 10^th^ centile. Many preterm infants demonstrate catch up in weight after hospital discharge, a time period equivalent to that in term born infants known to be strongly associated with later metabolic risk [8]. Children and adults born preterm demonstrate important differences in many aspects of their biology: cognitive outcome is worse [4], blood pressure is higher [9] and there is an increased prevalence of type II diabetes [10]. Other aspects remain less certain, for example data regarding the timing and progression of puberty after preterm birth are still conflicting [11,12]. Randomised controlled trials (RCTs) have shown that growth and nutrient intakes in early life in preterm infants affect metabolic outcomes in later childhood such as abnormal fat deposition, insulin sensitivity and vascular health [13-15]. Studies also show that early nutrition affects later cognitive outcome [16,17]. However, there are few longitudinal cohorts of preterm infants and there remain major uncertainties about how best to feed preterm infants. The risks and benefits of rapid growth promotion in both the pre- and post-discharge period, and in later childhood remain to be determined [18].

Along with accumulating evidence that early life exposures are key factors in determining later health, current lifestyle factors such as diet and physical exercise are also extremely important and provide an opportunity for targeted intervention. Recent efforts to improve nutritional status in adolescent children have proven difficult, and many of the factors that require change (e.g. diet, mealtimes, physical activity etc.) exist in a complex socio-cultural environment. In addition, although there is some evidence that short-term outcomes such as BMI (or fat mass index) can be modified, there are few data showing that insulin resistance or other markers of the metabolic syndrome are modifiable in children [19].

Determining the role of early life events in modulating the later risk of type 2 diabetes, obesity or hypertension require long term study as the underlying abnormal metabolic processes take decades before manifesting as disease. There are many challenges for longitudinal cohort studies, especially in high-risk cohorts such as children born preterm, including attritional losses over time [20] and changes in health care interventions that might limit generalizability to contemporary practice. Despite the enormous wealth of literature pertaining to animals, there are few prospective cohort studies in humans with detailed phenotypic characterization. Examples include the Avon Longitudinal Study of Parents and Children [21] and the Gateshead Millenium Study [22]. In some cohorts, follow up rates remain an issue, and most have only been tracked into childhood. Large-scale epidemiologic studies have provided a wealth of information on early growth patterns and long-term outcomes [23,24] but phenotypic characterisation of infant and childhood events are largely restricted to auxological parameters (i.e. weight, height and body mass index (BMI)) and there are few data with direct assessment of body composition. Whilst detailed phenotypic characterisation is present in certain longitudinal studies, most lack the granularity of information around delivery and in early postnatal life. Most regular birth cohorts also have preterm births under-represented, as difficult births are more likely to be omitted from sample collection due to other clinical priorities. Most adult cohorts are unable to determine relative differences in lean or fat mass accretion in early childhood, and data on early nutrient intake is largely limited to population estimates [25,26].

Much of the current data available from preterm infants is in cohorts recruited to studies in the 1980’s, an era pre-dating the widespread use of antenatal steroids and surfactant therapy. These two key perinatal practices have had dramatic effects on morbidity and mortality, and will have major effects on later outcomes. The Newcastle Preterm Birth Growth Study (Newcastle PTBGS) is a unique cohort of children born preterm in the late 1990’s. The major strengths are the detail of early nutritional and growth exposures, and the comprehensive assessment over time that includes growth, metabolic, neuro-cognitive, and body composition outcomes. The cohort also enables examination of outcomes from an RCT perspective, thereby avoiding some of the challenges of reverse causation and confounding.

Birthweight is a relatively crude measure of fat or lean mass, but despite this a clear relationship exists between birthweight and later lean mass and muscle strength [27,28]. This appears to be independent of muscle mass, suggesting that fetal and possibly infant growth permanently alter cellular function [29]. Intramyocellular and intrahepatic lipid accumulation alongside abdominal lipid depots compromise the ability to oxidise lipid, demonstrated by reduced muscle mitochondrial capacity and whole body cardiorespiratory fitness, both of which are key determinants of insulin sensitivity. Physical activity and inactivity are also important environmental modulators of insulin sensitivity through their direct and indirect influence on insulin action and also their role in weight maintenance. This strongly suggests that a comprehensive approach to outcome measurement in intervention trials is needed rather than focusing simply on weight loss or change in BMI. There are limited data on regional lipid deposition, oxidative capacity and glucose control across childhood, especially in those with detailed information of early growth and nutrient exposure.

To examine the relative importance of early growth and nutritional factors, and how these might confound or interact with later childhood factors requires study in cohorts with detailed phenotypic data. Genetic and/or epigenetic characterisation is likely to further improve understanding including possible mediating mechanisms (including molecular factors such as epigenetic processes) and establishing the direction of causality. In addition, there is a need to determine whether robust markers of later risk for type 2 diabetes, such as childhood insulin resistance (measured with fasting insulin and glucose and/or using an oral glucose tolerance test (OGTT), or intrahepatic/intramyocellular accumulation are modifiable. To do this, there is a need to first determine the current range of these parameters in high-risk groups and examine relationships with current measures of diet, physical activity and cardiorespiratory fitness, and establish the types of interventions that might be effective in decreasing the risk.

The aim of this study is to examine the associations between early life exposures in preterm infants and later metabolic outcomes and their mediators. We will follow up a cohort of preterm infants from the Newcastle PTBGS as teenagers to determine the presence of novel metabolic biomarkers and correlate these with lifestyle factors.

## Methods

### Study setting

The current study is embedded within the Newcastle PTBGS, which is a prospective cohort study based on two RCTs of varying nutrient intake in preterm infants: one study where the intervention was in the post-discharge period (standard versus nutrient enriched formula), and the second spanning the pre- and post-discharge period where differing protein, but iso-caloric formula intake densities, were fed [30,31]. All participants were born and recruited between 1993 and 1998 from a single tertiary neonatal centre, the Special Care Baby Unit at the Royal Victoria Infirmary in Newcastle upon Tyne. A control group of infants who were primarily breast-fed on hospital discharge, and some co-twins supplemented the two interventional cohorts. The infants of both RCTs and controls were combined to form the Newcastle PTBGS cohort.

The RCTs aimed to recruit infants who were born ≤34 weeks gestation and had a birthweight of ≤1750 g. In the first RCT, referred to as the ‘growth study’ , infants were randomised to one of two formulae on discharge from hospital. They were either fed (A) a preterm infant formula from discharge to 6 months post-term, (B) a standard term formula from discharge to 6 months or (C) the preterm formula from discharge to term and then the standard term formula until 6 months (‘crossover’ group). Preterm formula had an energy density of 80 kcal and 2.2 g protein, and term formula was 67 kcal and 1.6 g protein per 100 mL [32]. Further details of the nutrient composition of the formula are provided elsewhere [32]. A control group of breastfed babies was also included. Infants were seen at the outpatient clinic fortnightly between discharge and term and monthly between term and 6 months corrected age. At each clinic visit, milk intake, auxology (length, weight, mid-arm circumference, MAC, occipito-frontal head circumference, OFC) and serum biochemistries were determined. Formula milk intake was determined by providing pre-weighed ready to feed formula, and re-weighing used bottles. Body composition was assessed at different time points including hospital discharge, term, 12 weeks, 6 months, and 12 months using dual energy x-ray absorptiometry (DXA) scans. Auxological measures were repeated at 18 and 24 months. Bayley Scales of Infant Development (BSID) mental development index (MDI) and psychomotor development index (PDI) tests were assessed at 18 months [30].

In the second RCT, the ‘protein study’ , 77 infants were randomised to one of three preterm formulas that were iso-caloric (80 kcal/100 mL), but differed in protein density (A - 3.3 g, B - 3.0 g, and C - 2.7 g per 100 kcal) [31]. Infants were recruited once full enteral feeding had been successfully established and they continued on the trial formula post-discharge until 12 weeks post-term. Milk intake, auxology and serum biochemistries were determined weekly during inpatient stay, and every 4 weeks until 12 weeks. Auxology was repeated 6 monthly until 24 months. Body composition was assessed using DXA at discharge and at 12 weeks.

In both studies, a social questionnaire addressing aspects of family structure, parental employment, education history, smoking etc., was completed prior to hospital discharge. Although the inclusion criteria for randomisation into the two RCTs was a gestational age of ≤34 weeks, 1 set of low birthweight triplets of 36 weeks gestation were included as breastfeeding controls, along with siblings of eligible infants whose birthweight was >1750 g. Because of a small time overlap in the recruitment period for the two RCTs (randomised infants could only join one RCT) there were 4 breast fed control infants who were initially included in study reports for both studies, but thereafter are only included once in the combined cohort. Mean gestation for the cohort (n = 247) was 30.9 weeks (range 24.7-36.4 weeks) and birthweight 1392 g (range 690-2200 g). One hundred and twenty five were male. There were no significant differences in gestation or weight between boys and girls.

The Newcastle PTBGS has previously been followed up on three occasions since the initial randomised controlled trials (see Table 1). The current study, GROWMORE, will be phase four of follow-up [33].

**Table 1:** Data collected during follow-up phases of the Newcastle preterm birth growth study

**Phase 1- Growth and Protein study RCTs**	**Phase 2- Cognitive/Behavioural follow-up at 10 years**	**Phase 3- Early adolescent follow-up**	**Phase 4- Late adolescent follow-up**
**(n = 247)**	**(n = 92 from 113 in growth study)**	**(n = 153 from 247 in RCTs)**	**(n = 60 from 153 in phase 3)**
**Auxology:**		**Auxology:**	**Auxology:**
Length*, weight, OFC at birth, discharge, term, 1,2,3,6,12,18,24 months corrected age	Wechsler Intelligence Scale for Children version 3 (WISC III) including Wechsler Objective Numerical Dimensions (WOND), Wechsler Objective Reading Dimensions (WORD) and Wechsler Objective Language Dimensions (WOLD). Achenbach Child Behaviour Checklist. Vineland adaptive behavior scales (n = 113)	Height, weight, OFC, abdominal circumference, waist/hip ratio	Height, weight, waist circumference, blood pressure. Self assessment of pubertal status
		Blood pressure	
		Self-assessment of pubertal status	
		(n = 153)	
**Bloods:**		**Bloods:**	**Bloods:**
Serum Biochemistry weekly till term then 1,2,3 months		Short oral glucose tolerance test	2 hour glucose tolerance test, 25-OHD, lipid profile, liver function, sex hormones, RNA, cellular DNA and serum to store
		Fasting Lipids: cholesterol, triglycerides	
Leptin, adiponectin, insulin and IGF1 (n = 70 subset)		Leptin, adiponectin	
		(n = 109)	
**Body composition:**		**Body composition:**	**Body composition:**
DXA at discharge and/or term, 3,6,12 months (6 and 12 months only growth study)		DXA scan and bio-impedance	BODPOD
		(n = 139)	IHL and IMCL deposition using ^1^H magnetic resonance spectroscopy
Social questionnaire	Socioeconomic deprivation score	Social and family history	SCRAN-24 dietary information
			Actigraph data
			Intervention and feedback questionnaires
BSID II MDI and PDI at 18 m (Growth study n = 113)			

*Length not available pre-discharge for most.

### GROWMORE study protocol

#### Hypotheses

1. Muscle oxidative capacity determined by ^31^P MRS and cardiorespiratory fitness are related to measures of physical activity, physical inactivity, insulin resistance and Vitamin D status in adolescence.

2. Auxological measures (weight, height, OFC and skinfold) provide a reliable measure of adiposity compared to non-invasive body composition assessment using air displacement plethysmography (BODPOD™).

3. Intra-hepatic or intra-myocellular lipid accumulation is related to markers of insulin resistance, raised triacylglycerol and measured body fat in adolescents

4. Body composition and metabolic outcomes in adolescence are related to patterns of infant growth, body composition and nutritional intakes.

#### Power calculation

Determining an appropriate sample size for this study is difficult given there are multiple outcome variables, including some exploratory, being investigated. Using results from phase three of this study, we know that insulin sensitivity is strongly associated with fat mass index. Using the effect size from our data of r^2^ = 0.12, with 80% power at the 5% level of significance we estimated that we would need ~60 patients to be able to detect a significant difference. Previous work looking at mitochondrial oxidative function in the Magnetic Resonance centre required a sample size of only 10 patients to determine a 10% reduction in τ_1/2_ PCr [34]. Therefore we have chosen to study 60 young people, as this will ensure an adequate sample size for two of the main outcome variables.

#### Current study population and recruitment

All children who participated in phase 3 (n = 153) will be approached, via mail, after verifying postal address through their general practitioner. We will approach the young people sequentially starting with the oldest, but no other active selection process will be used. A recruitment letter, explaining the study and assessments in detail will be sent to all parents whose children fit the above criteria and a simplified patient information sheet sent to the young person. Informed, written consent will be obtained from parents and where appropriate, participants, before participation.

#### Study design

**Figure 1:**
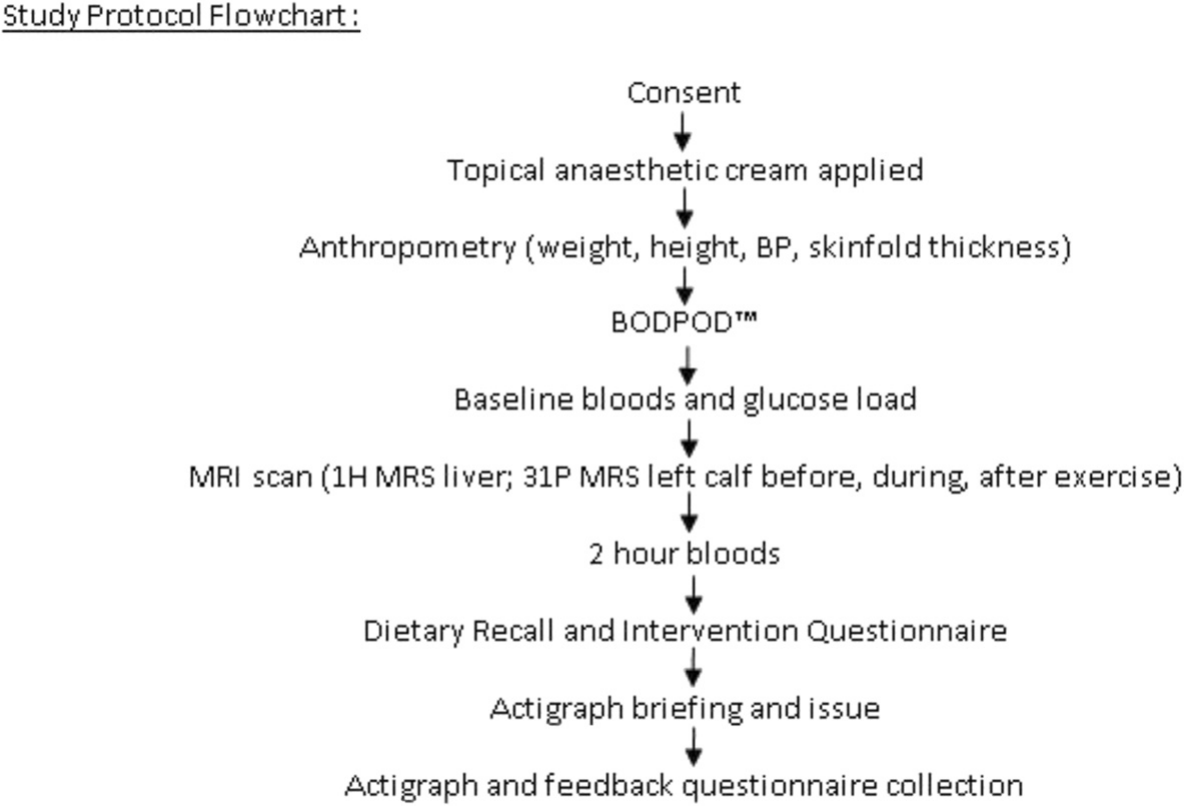
The study protocol flowchart.

The same researcher (RT) will carry out all assessments. Figure 1 shows the study protocol flowchart. All initial assessments will be carried out during a single morning visit to the Newcastle Magnetic Resonance Imaging Centre. Participants will attend fasted. Once the initial assessment is finished (within 4 hours), the pre-programmed accelerometers (Actigraphs) will be given to each young person to wear for 3 days and a feedback questionnaire given. The Actigraph and questionnaires will then be returned and data analysed.

We will use the following methods during assessment:

1. **Anthropometry**

• Height will be measured to the nearest 0.1 cm, using a telescopic stadiometer (SECA, Birmingham, UK)

• Weight will be taken to the nearest 0.1 kg from the BODPOD**™ (**Cosmed, USA). BMI will be calculated using these measurements. Standard deviation scores will be calculated for height, weight and BMI using standard reference data.

• Waist circumference will be measured using a measuring tape (CTO Group, China).

• Weight and height standard deviation scores, adjusted for sex and age will be calculated using the LMS calculator and British 1990 growth reference standards [35,36].

• Blood pressure will be taken from the right arm, using an automated sphygmomanometer. (Carescape vital signs monitor, GE Healthcare systems, UK)

2. **Body composition**

Initially, Holtain calipers (Crymych, UK) will be used to measure skinfold thickness in four areas- biceps, triceps, subscapular and suprailiac. These will be used to generate an estimate of body density [37,38]. A second estimate of body composition will also be made using BODPOD™ [39,40]. The BODPOD uses air displacement plethysmography and has been validated for use in children [41]. BODPOD measures body mass and volume by calculating differences in volume of the participants compared to calibration volumes (age-dependent) of the participant sitting inside the BODPOD, thus allowing body density to be calculated. Once overall body density is determined, the relative proportions of lean and fat body mass can be calculated.

3. **Biochemistry and insulin sensitivity**

All participants will attend fasted. Baseline bloods will be taken (for lipid profile, liver function, glucose, insulin, sex hormones, RNA, cellular DNA and serum to store) and then a standardised fasting-state oral glucose tolerance test will be performed [42]. Insulin sensitivity and resistance will be measured using the Homeostasis Model Assessment (HOMA) method.

Sinha et al. [34] have also demonstrated a clear association between skeletal muscle mitochondrial oxidative function and serum vitamin D levels in patients with Growth Hormone deficiency using the same 31-P MRS exercise protocol as we will use. In order to ensure we can control our data for any influence of vitamin D status, frozen serum will be assayed using a Liquid Chromatography-Tandem Mass Spectrometry (LC-MS/MS) method to obtain a 25-OH Vitamin D quantification.

4. **Epigenetic sampling**

We will seek consent to store any spare serum and DNA (extracted from blood and saliva) for future analysis [43,44]. These samples will be subject to the Human Tissue Act 2004 [45] and held under license (issue number 12534, held by Newcastle Biomedicine Biobank). DNA will be extracted at the Institute of Genetic Medicine, Newcastle University, Newcastle-upon –Tyne using standard techniques [46-48].

5. **Measurement of intra-hepatic lipid**

Intra-hepatic lipid (IHL) and Intra-myocellular lipid (IMCL) deposition will be determined using ^1^H magnetic resonance spectroscopy. Details of the planned MR sequences will be similar to those used in our previous work [49]. Percentage of intrahepatic lipid will be calculated using the % of CH_2_ lipid peak signal amplitude, relative to the water peak signal amplitude [50-52].

6. **Measurement of mitochondrial oxidative function**

**Figure 2:**
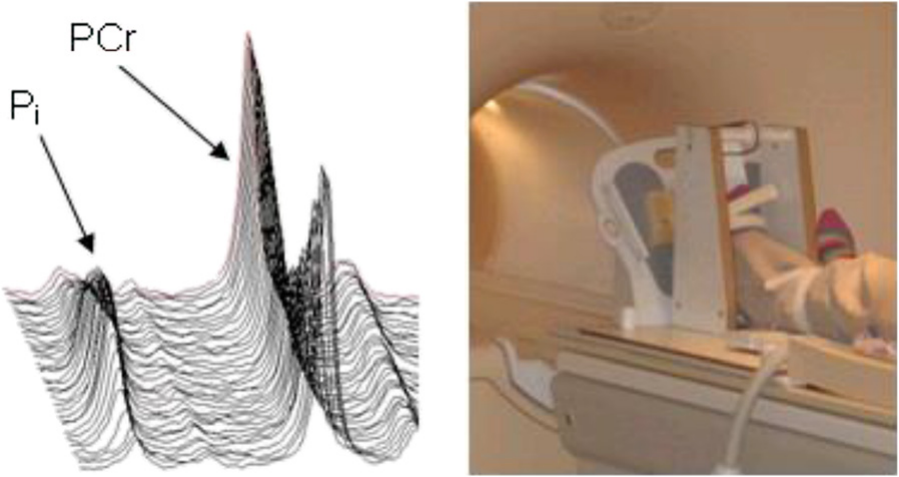
**Typical 31-P spectra generated during exercise within the MR scanner and the equipment that will be used for the study **[55]**.**

We will use custom-built exercise apparatus to allow each participant to perform a standardised exercise protocol as previously described by members of our group [34,53,54], consisting of plantar flexion against fixed resistance during which time 31 P-MRS will be used to assess mitochondrial oxidative function (see Figure 2. In order to generate the ^31^P spectra, a 10 cm diameter ^31^P surface coil will be placed over the calf muscles, centred on the widest part of the gastrocnemius/soleus complex, for transmission and reception of the signal. After calf muscle group maximum voluntary contraction (MVC) has been determined for each participant, the exercise apparatus will be set to provide resistance of 35% of MVC, and ^31^P spectra will be recorded over a three minute time window to assess resting mitochondrial function. This will be immediately followed by a three-minute exercise period during which the participants will be required to perform plantar flexion against the set resistance at a frequency of 0.5 Hz and then a further rest period. MR phosphorous spectra will be collected every 10 seconds during the protocol using adiabatic One-Dimensional Image-Selected In-vivo Spectroscopy (1D-ISIS). This protocol has been shown to robustly measure mitochondrial oxidative function during recovery from exercise [34,53].

The phosphorus spectra will be analysed in batches using the Advanced Method for Accurate, Robust and Efficient Spectral fitting (AMARES) algorithm [56] and from this quantifications of phosphocreatine (PCr), inorganic phosphate (Pi) and pH throughout the protocol will be obtained. The data will then be processed to calculate the various components of oxidative metabolism as described by Kemp and Radda [57]. The primary outcome measure will be the half-time of PCr recovery [53] (τ_1/2_ PCr). This recovery period is used to measure mitochondrial oxidative phosphorylation ‘capacity.’ In addition, the half-time of ADP (τ_1/2_ ADP) recovery will be measured as it provides a similar measure to τ_1/2_ PCr but may be more resistant to heterogenous pH responses at the end of exercise.

7. **Physical activity levels**

Physical activity levels over a 24-hour period will be measured using an Actigraph **™**accelerometer (Actigraph, Pensacola, FL). The Actigraph is a small and lightweight monitor that will be worn on the right hip during waking hours for three days. The Actigraph has high validity, reliability, and low reactivity in children [58] and has been used successfully in several paediatric physical activity studies [59]. The accelerometers will be set to record data in 10-second epochs. Analysis of the activity data will be performed using Actilife program (v5, MTI, Pensacola, USA). A written diary will also be kept to monitor periods of non-wear. From the data gained we will calculate the time spent in sedentary or moderate to vigorous physical activity (MVPA). MVPA has been chosen rather than looking individually at different activity levels: it has been shown in a term born group of children in north east England that MVPA seems to be most closely associated with body habitus change when followed longitudinally [60].

8. **Diet and nutritional assessment**

The SCRAN-24 dietary recall program will be used to determine each participant’s food/drink intake over the previous 24 hours. (Human Nutrition Research Centre, Newcastle University) [61]. SCRAN-24 uses a series of steps to determine the times of food/drink intake over the 24 hours prior to using the program. It asks the user to list chronologically the items of food or drink consumed in the 24-hour period under a series of headings (breakfast, mid-morning snack, lunch etc.). It then breaks down each intake into their component parts. For the majority of foodstuffs (98% of those regularly consumed by children in the north east of England), the programme contains a listed item. Non-listed items have to be entered in free-text. Once the items are selected for the full 24-hour period the program then revisits each episode of food intake and prompts the participant to assess and log portion size based on a series of photo-based choices. Pre-programmed nutritional information for each listed item, by portion size is allocated to each item. A final step prompts the user to try to link food and drink intake with activity through the day and highlights any times of the day during which nothing was ingested as particular areas to focus on. This is done to try to improve recall and thus improve the sensitivity of the diary. SCRAN-24 has been validated for use in people in the north east of England aged 11 years of age or over.

9. **Questionnaires**

Intervention questionnaires will also be given to the participants, asking opinions on different potential structured activities within a programme designed to encourage a healthier lifestyle in children and their families. This data will help to inform potential future interventional studies about which interventions might be most accepted by a similar teenage cohort.

Following the assessment we will give each young person a feedback questionnaire to fill in to assess their experience of the study assessment day and their perceived acceptability of the assessment methods. By reviewing how many participants accept, decline or are unwilling/unable to complete particular assessments we will gauge their usefulness for future trials.

The study has been approved by the Sunderland Research Ethics Committee and the Research and Development Department of Newcastle Hospitals NHS Trust.

#### Statistical analysis

Data will be analysed using Stata (v11, StatCorp LP, Texas, USA). Mann–Whitney U or t tests will be used to demonstrate that the follow-up sample is broadly representative of the original cohort for key variables such as gestation and birth weight. Correlation analysis will be used to examine the relationships between the different measures of body composition and the MR measures of lipid deposition and to examine the interaction between vitamin D and oxidative capacity by ^31^P MRS and cardiorespiratory fitness. To assess associations between infant and childhood growth and subsequent metabolic outcomes, we will develop separate linear regression analysis models using insulin sensitivity and body composition (fat mass index and lean mass index) as the dependent variables. Half-time of PCr recovery (τ_1/2_ PCr) will be used as dependent variable for regression models examining muscle mitochondrial function in relation to insulin sensitivity, directly measured exercise, body composition and vitamin D levels (as independent variables). In all multiple linear regression models adjustment will be made for potential confounding factors such as sex, gestation, age at follow-up, birthweight z-score, infant growth velocity, post discharge feeding pubertal status and whether mechanical ventilation was required in the neonatal period (which we used as a proxy for illness severity). Bland Altman analysis will be used to assess agreement between the different techniques used to assess body composition.

## Discussion

### Key findings from previous phases of the Newcastle PTBGS

The growth study showed significantly improved growth in those receiving the preterm formula although the growth advantage was greater for boys than girls [30]. At 18 months corrected age, male infants fed preterm formula were 1 kg heavier, 2 cm longer, and had 1 cm greater head circumference than those fed the term formula. Increased weight gain primarily reflected an increase in lean mass as shown by DXA, consistent with the idea that the preterm formula more closely met protein-energy needs in rapidly growing preterm male infants [62]. However, no RCT group differences were demonstrated in developmental outcome (BSID) at 18 months despite differences in head growth earlier on. This may reflect the fact that infants on the term formula appeared to up-regulate their intake volumes so caloric intake was similar between the groups [63]. Differences in growth are likely then to primarily reflect increases in protein intake (because the protein: energy ratio was also different between the two formulae). In addition, the greatest differences in nutrient accretion occurred between discharge and term, with only relatively small differences in growth thereafter. This suggests that the timing of dietary intervention may be critical. In term populations increased weight gain in the first few months i.e. in the same time window as preterm infants post-discharge, is associated with later risk of obesity [8].

The protein study did not show any significant differences in growth or body composition between isocaloric formula with differing protein concentrations of 2.7 g, 3 g or 3.3 g/100 Kcal. Infants were recruited at ~34 weeks corrected gestation, and continued on the trial formula until 12 weeks post term [31]. There was a non-significant trend in boys to increased growth (higher weight, length and OFC gain) pre-discharge on the higher protein intakes, but there were no group differences at 12 weeks post-term in auxology or body composition. Protein intakes were closely paralleled by changes in serum urea nitrogen and these differed between the groups. The trial may have been relatively under-powered to show effects of higher protein intake pre-discharge especially as the protein densities only differed by 10-20%. Post-discharge there appear to be no advantage to protein densities higher than 2.7 g/100 kcal.

Data on the relationship between early nutrient intakes, growth patterns and subsequent measures of obesity, insulin sensitivity, cognition and bone mineral density are being further analysed [64]. Blood and saliva samples collected at adolescent visits were analysed for epigenetic correlates of early growth patterns. Change in weight standard deviation score between term and 12 weeks post-term was used to determine patterns of catch up growth post-discharge. Exploratory analyses using microarrays identified differentially expressed genes in whole blood from children who had either “slow” (n = 10) or “rapid” (n = 10) early postnatal growth [46,48]. Methylation analyses in 121 samples from the Newcastle PTBGS identified several potential candidate genes, one of which (*TACSTD2*) was analysed in relation to adolescent body composition. *TACSTD2* expression was inversely correlated with DNA methylation, and both measures were associated with fat mass (FM) [46]. However, the lack of an association between a methylation proxy single nucleotide polymorphism and FM suggested that reverse causation or confounding might explain the association.

In a further study, gene expression analysis undertaken in the PTBGS children with slow or rapid weight gain was used to generate a panel of differentially expressed genes for DNA methylation analysis undertaken in a cohort of term children with samples collected as part of the ALSPAC cohort [47]. Of the 29 differentially expressed genes, there were associations between methylation and at least one index of body composition (BMI, FM, lean mass, height) in around one third of them in the comparator cohort. However, in only one of the 9/29 genes did the association remain after correction for multiple testing. This was an association between the *ALPL* gene, encoding the enzyme alkaline phosphatase, and height, a relationship that has biological plausibility.

The current phase of this study will provide a unique insight into the metabolism of teenagers born preterm. It will allow direct correlation of clinical and research measures of body composition with each other, and with insulin sensitivity, which may be important for future health. It will allow comparison of directly measured physical activity and muscle recovery capacity in the cohort as well as measures of diet. It will also provide an invaluable opportunity to further examine whether associations between a variety of early growth and nutritional exposures and later phenotype, may be modulated by epigenetic mechanisms.

The Newcastle PTBGS is a unique resource in individuals at high risk of the metabolic syndrome who may have experienced a range of adverse exposures in pre- and postnatal life. Detailed phenotypic measures can be correlated with early life nutritional and growth exposures, and the relationship adjusted for contemporary lifestyle factors. Precise measures of early growth, appetite, blood-based biomarkers, and nutrient intake, and the randomised nature of the interventions along with careful tracking over time mean the Newcastle PTBGS will provide unique insights into lifecourse epidemiology.

## Abbreviations

ADP: Adenosine diphosphate; BMI: Body mass index; BSID: Bayley scales of infant development; DXA: Dual X ray absorptiometry; FM: Fat mass; HOMA: Homeostasis model assessment; IHL: Intrahepatic lipid; IMCL: Intramyocellular lipid; MAC: Mid arm circumference; MDI: Mental development index; MRS: Magnetic resonance spectroscopy; MVPA: Moderate to vigorous physical activity; OFC: Occipito-frontal head circumference; PCr: Phosphocreatine; PDI: Psychomotor development index; PTBGS: Preterm birth growth study; RCT: Randomised controlled trial.

## Competing interests

The authors declare that they have no competing interests.

## Authors’ contributions

CW wrote the manuscript and performed analysis of phases 2 and 3 of the study. RT developed the study protocol and will perform the assessments and analysis. MK designed and performed assessments in phase 3. CR, MT, KH, TC helped develop and provide expertise in study design. MP has provided statistical expertise throughout phases 2 and 3 of the study and helped with statistical analysis of this phase. RC helped design and carry out the initial study. NE established the PTBGS cohort, performed assessments in phase 1 and has coordinated all phases. All authors have read and approved the final manuscript.

## Pre-publication history

The pre-publication history for this paper can be accessed here:

http://www.biomedcentral.com/1471-2431/13/213/prepub
